# Supplementary material for: Risperidone Exacerbates Glucose Intolerance, Nonalcoholic Fatty Liver Disease, and Renal Impairment in Obese Mice
Source: Int J Mol Sci. 2021 Jan 2;22(1):409. doi: 10.3390/ijms22010409 (PMC7795724; doi:10.3390/ijms22010409)
Supplement: Supplementary file 1 [file ijms-22-00409-s001.pdf]

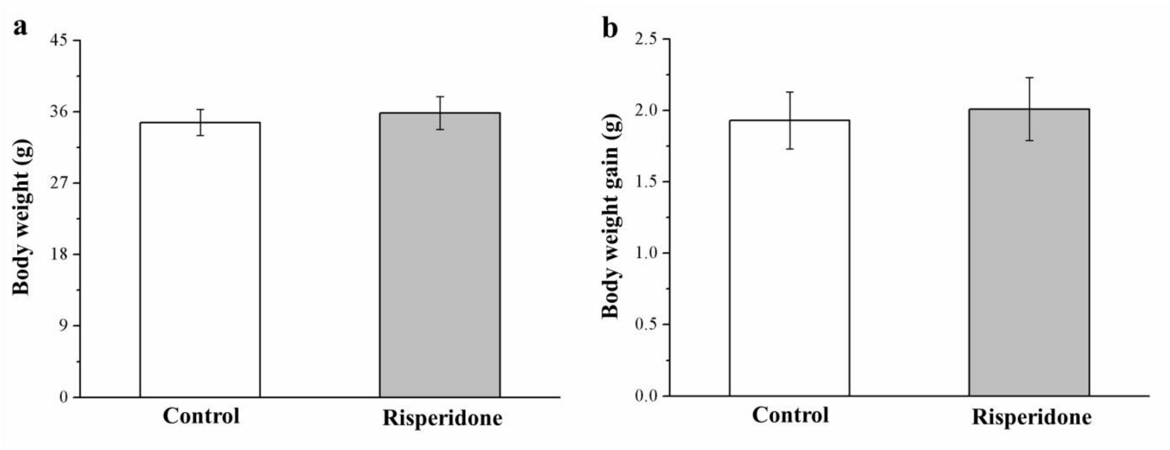

Figure S1. Changes in body weight and weekly body weight gain measured in control and 1 mg/kg/day risperidone-treated SD-fed mice over the 56-day period.

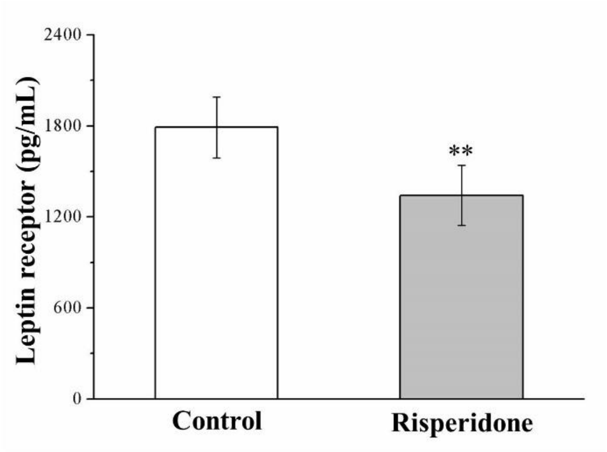

Figure S2. Changes in serum soluble leptin receptor levels measured in control and 1 mg/kg/day risperidone-treated obese mice over the 56-day period. ***P* < 0.01.

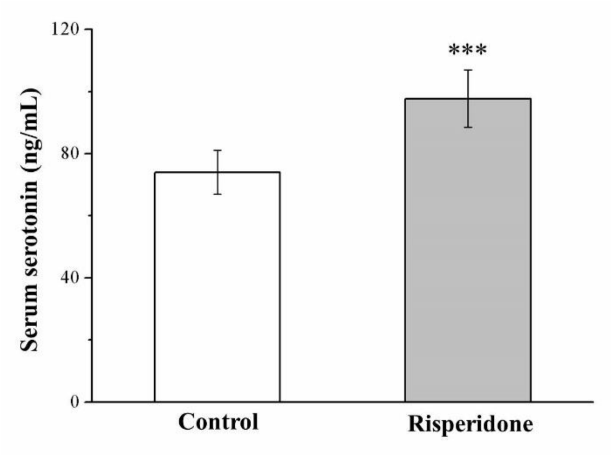

Figure S3. Changes in serum serotonin levels measured in control and 1 mg/kg/day risperidone-treated obese mice over the 56-day period. ***$P < 0.001$.

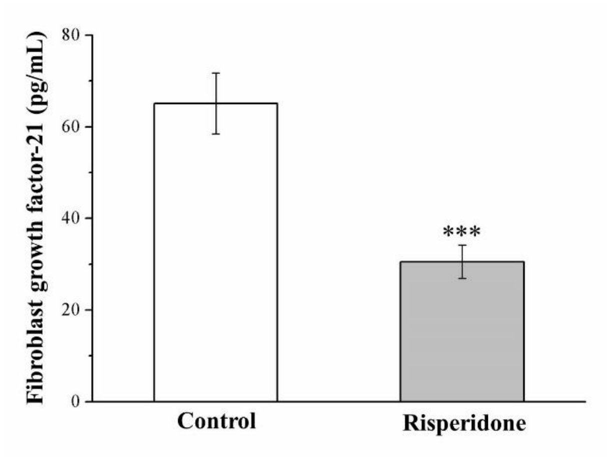

Figure S4. Changes in serum FGF-21 levels measured in control and 1 mg/kg/day risperidone-treated obese mice over a 56-day period. ***$P < 0.001$.

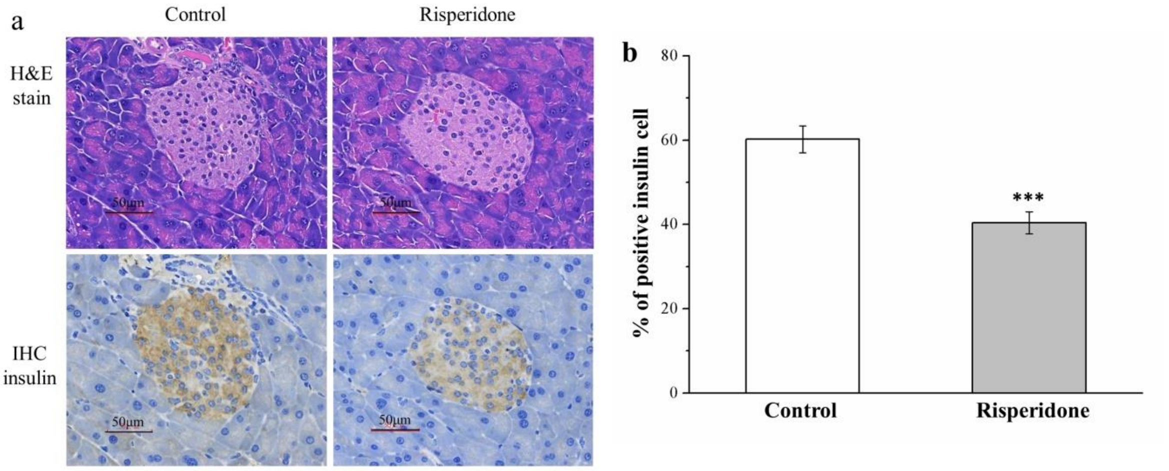

Figure S5. Changes in (a) islet morphology (H&E stain and immunohistochemical stain; 200× magnification) and (b) β-cell percentage measured in control and 1 mg/kg/day risperidone-treated obese mice over a 56-day period. $^{***}P < 0.001$.

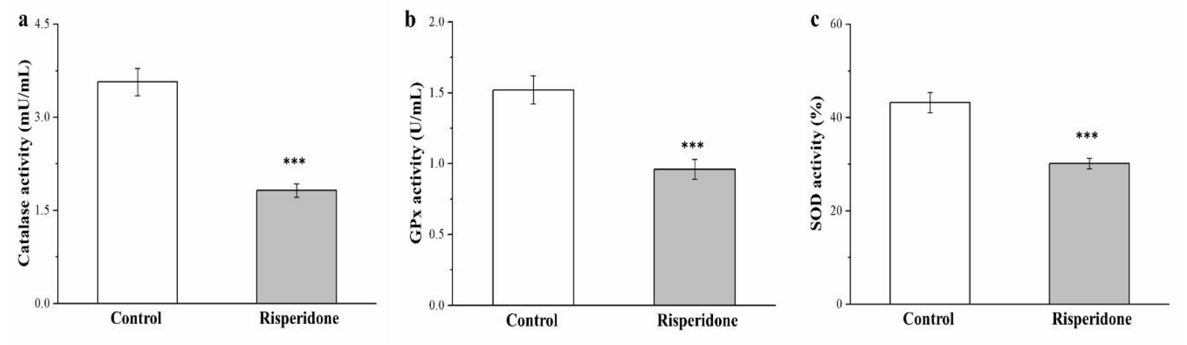

Figure S6. Changes in pancreatic (a) catalase, (b) GPx, and (c) SOD activities in the 1 mg/kg/day risperidone treatment and control groups over the 56-day treatment period. ***$P < 0.001$.

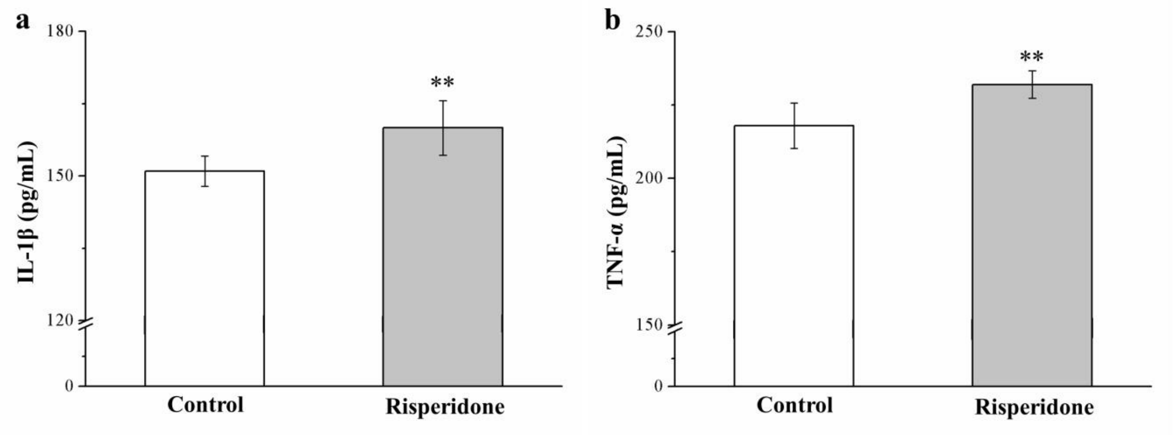

Figure S7. Changes in the serum levels of (a) TNF-α and (b) IL-1β measured in control and 1 mg/kg/day risperidone-treated obese mice over a 56-day period. **$P < 0.01$.

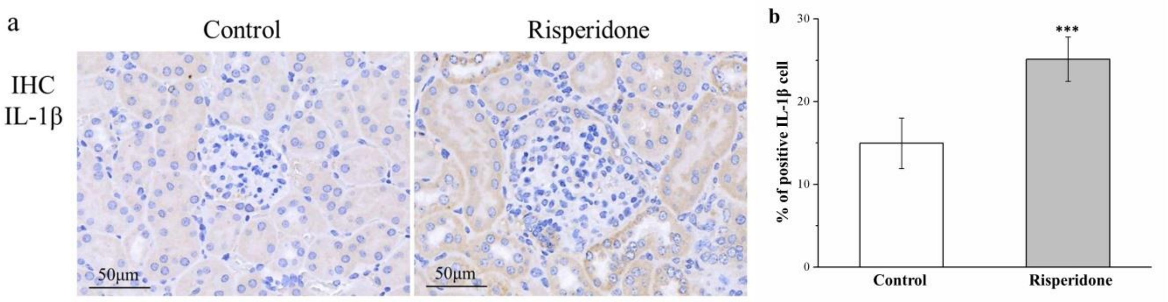

Figure S8. Changes in (a) immunohistochemical stain (200× magnification) and (b) kidney levels of IL-1β measured in control and 1 mg/kg/day risperidone-treated obese mice over a 56-day period. ***$P < 0.001$.
